# Production of Mesoglycan/PCL Based Composites through Supercritical Impregnation

**DOI:** 10.3390/molecules27185800

**Published:** 2022-09-07

**Authors:** Sara Liparoti, Stefania Mottola, Gianluca Viscusi, Raffaella Belvedere, Antonello Petrella, Giuliana Gorrasi, Roberto Pantani, Iolanda De Marco

**Affiliations:** 1 Department of Industrial Engineering, University of Salerno, Via Giovanni Paolo II, 132, 84084 Fisciano, SA, Italy; 2 Department of Pharmacy, University of Salerno, Via Giovanni Paolo II, 132, 84084 Fisciano, SA, Italy; 3 Research Centre for Biomaterials BIONAM, University of Salerno, Via Giovanni Paolo II, 132, 84084 Fisciano, SA, Italy

**Keywords:** supercritical carbon dioxide, polymer blend, compression molding, electrospinning, drug release

## Abstract

The development of targeted therapies for wound repair is knowing a growing interest due to the increasing aging of the population and the incidence of chronic pathologies, mainly pressure ulcers. Among molecules recruiting cell populations and promoting the formation of new vital tissue, sodium mesoglycan (MSG) has been proven to be effective in wound healing. In this work, MSG impregnation of polymer matrices has been attempted by a supercritical carbon dioxide-based process. Polymeric matrices are composed of polycaprolactone blends, where water-soluble polymers, polyethylene glycol, polyvinyl pyrrolidone, gelatin, and thermoplastic starch, have been employed to modulate the MSG release, making the devices potentially suitable for topical administrations. Two different techniques have been used to obtain the films: the first one is compression molding, producing compact and continuous structures, and the second one is electrospinning, producing membrane-like designs. A higher amount of MSG can be loaded into the polymeric matrix in the membrane-like structures since, in these films, the impregnation process is faster than in the case of compression molded films, where the carbon dioxide has firstly diffused and then released the active molecule. The type of water-soluble polymer influences the drug release rate: the blend polycaprolactone-gelatin gives a prolonged release potentially suitable for topical administration.

## 1. Introduction

Wound management has been a big issue that costs billions of dollars worldwide. The increasing aging of the population and the incidence of chronic pathologies make the development of targeted therapies for wound repair, particularly advanced dressings, mandatory [1,2]. Wound dressings are a kind of material that covers wounds to protect them from damage and promote their healing [3,4]. Wound dressings can be classified by their forms into gauze [5,6,7,8,9,10,11,12], hydrogels [13,14,15,16,17,18], foams [19,20,21], and others [22,23]. Conventional wound dressings are designed to control hemorrhage and promote wound healing. For this purpose, a wound dressing must protect the wound from secondary injuries, keep the moisture of the wound site, facilitate the removal of excess exudate, and prevent infection [24,25]. Furthermore, wound dressing has to promote healing by realizing active molecules on the wound site. In recent years, topical therapy involves the use of bio-inducers inducing the cell release macromolecules of extracellular matrix (ECM), recruiting cell populations essential for the formation of new vital tissue. Among the molecules keeping these criteria [2], sodium mesoglycan (MSG) has been proven to be effective in the tissue regeneration [26,27].

It is extracted from porcine intestinal mucosa and composed of natural glycosaminoglycans, heparan-, dermatan- and chondroitin-sulfate, and low molecular weight heparin. It is approved for treating vascular diseases with associated thrombotic risks. Mesoglycan can enhance re-epithelialization and granulation processes, acting on human epidermal keratinocytes and dermal fibroblasts, favoring angiogenesis. Mesoglycan was proven to be efficient for the treatment of patients affected by pressure ulcers [28]. Moreover, it has been proven that MSG can promote the new blood vessels formation and an anti-inflammatory response [27,29]. For these reasons MSG is considered an attractive therapeutic agent in the treatment of skin wound healing since it has been proven to be able to act in the main phases of this process, from injury to remodeling [3,29].

Although there is great interest in wound dressing devices, conventional devices present some drawbacks. For instance, most of them cannot adjust their properties with the wound condition changing. Meanwhile, developing wound dressing for specific parts of the body, such as the joints and necks that experience high stretch stress, is challenging. Additionally, the pain caused by changing wound dressings has not been heeded. Thus, developing innovative devices capable of prolonging the release of molecules promoting wound healing is still a challenge. Natural polymers, such as alginate, gelatin, etc., are widely used to obtain films for skin regeneration dressings [30,31]. However, they are water-soluble, causing prolonged drug administration [30]. Synthetic polymers showing degradation within a specific time span after dressing a wound may provide provisional support for molecules promoting healing [32]. Few biodegradable polymers, such as polylactide (PLA), polyglycolide, and their copolymers semi-crystalline polycaprolactone (PCL), are used for biomedical applications [33]. Among the various biodegradable polyesters, PCL is the most promising material for wound healing and tissue engineering [34,35].

Loading the active molecule in the polymeric film in a sustainable manner is challenging. The conventional methods for obtaining topical devices promoting wound healing show high organic solvent residues and low drug levels incorporated into the polymeric matrix, thus limited loading efficiency. The impregnation of an active molecule into a polymeric matrix can be attempted using supercritical carbon dioxide (scCO_2_) [36,37]. The scCO_2_ solubilizes the active principle to be impregnated thanks to its high solvent power. It acts as plasticizing and swelling agent for polymers [37], allowing a rapid penetration into matrices and the formation of a porous structure. The scCO_2_ has been recently proposed for obtaining medical devices for wound healing [38,39,40].

In this work, the impregnation of PCL blends with MSG by scCO_2_ has been attempted. Particularly, blends of PCL and water-soluble polymers, such as polyethylene glycol, polyvinyl pyrrolidone, gelatin, and thermoplastic starch, have been used for modulating drug release. The scCO_2_-based process has been used to impregnate two kinds of films: the first ones, obtained by compression molding, show a continuous and compact structure; the second ones are membranes composed of electrospun fibers. The different structure of the starting materials is expected to influence the release of the active compound.

## 2. Results and Discussion

### 2.1. Compression Molded Films

**Figure 1:**
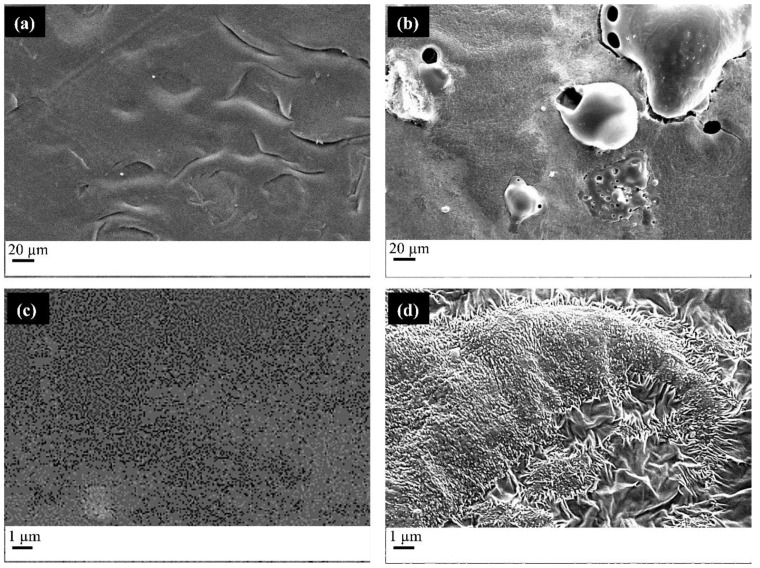
FESEM micrographs of the (**a**) PCL/starch film obtained by compression molding (image at low magnification), (**b**) PCL/starch film impregnated with MSG (image at low magnification), (**c**) PCL/starch film obtained by compression molding (image at high magnification), (**d**) PCL/starch film impregnated with MSG (image at high magnification).

PCL/Starch films were produced by compression molding and then impregnated with MSG. In Figure 1, the SEM images of the different samples are reported. Figure 1a,c show the film of PCL/Starch with a weight ratio of 80/20. Figure 1b,d show the film after the impregnation process.

The films in Figure 1a,c show a homogeneous and continuous structure, proper of the parts obtained by compression molding, with the presence of some cracks, due to the immiscibility between the polymers constituting the blend. After the scCO_2_ impregnation process, porosities can be detected. As it is possible to observe from Figure 1b, pore dimension and distribution on the surface are not homogeneous for the presence of continuous areas and pores with wide dimension distribution. The observed morphology probably is the result of a non-homogeneous foaming process induced by the presence of polymers showing a different behavior in the presence of scCO_2_. This shows lower solubility in starch [41] than PCL [42]. The blend morphology is controlled by parameters such as the nature of the polymers (interfacial energy and viscosity ratio), the blend’s composition, and processing conditions. Coalescence phenomena often take place, giving rise to the formation of a dispersed phase composed of the minor component. In our case, starch is the minor component and forms a dispersed phase [43]. During the impregnation, each polymer reacts in different ways to the presence of scCO_2_: PCL allows for pore formation, whereas starch does not form pores. As a result, the pore distribution is not homogeneous. It is also important to consider that the foamability of a blend is strongly determined by the solubility, diffusivity and elongational viscosity of the polymers and that the nucleation and growth of pores occur not only in the polymer matrix, but also at the interface of the two polymers.

Figure 1d shows the presence of small crystals, in the form of needles, related to the presence of MSG, on the whole surface. Their presence can be ascribed to two phenomena: the first is related to the impregnation process, and the second is associated with the depressurizing step that induces the decrease of MSG solubility in CO_2_ and its precipitation in the form of crystals. Concerning the first phenomenon, it can be hypothesized that MSG solubilized in scCO_2_ can mainly diffuse near to the polymer surface, which leads to the formation of crystals partially embedded in the polymer matrix. The second phenomenon occurs in all the analyzed conditions since the decrease of pressure induces a reduction of the active compound solubility. In this case, molecules precipitate in the form of needles, which do not adhere to the polymer matrix.

**Figure 2:**
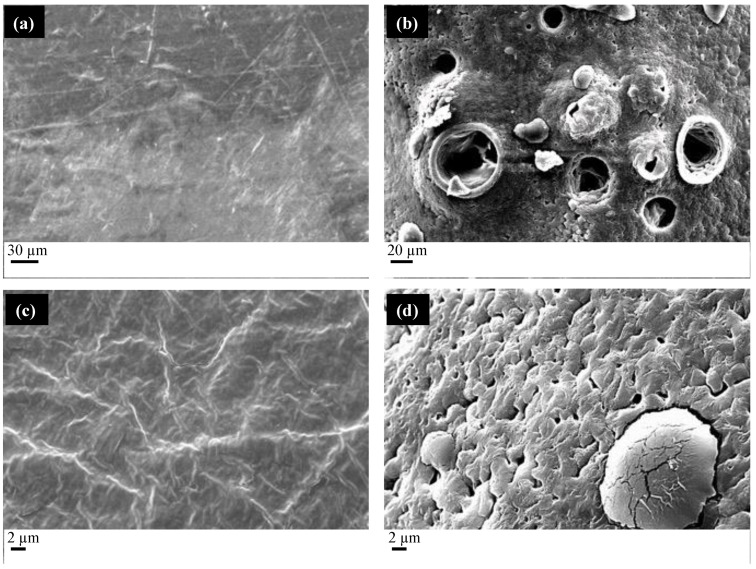
FESEM micrographs of the (**a**) PCL/PEG film obtained by compression molding, (**b**) the film impregnated with MSG through scCO_2_, (**c**) an enlargement of the PCL/PEG film obtained by compression molding, (**d**) an enlargement of the scCO_2_ impregnated MSG PLC/PEG film.

Figure 2a shows the films produced by compression molding with a PCL/PEG weight ratio of 80/20. Figure 2b shows the film after the impregnation with MSG.

The compression-molded film appears continuous and compact, as in the case of PCL/starch films. The scCO_2_ impregnation (see Figure 2b) process induces the formation of a porous structure, characterized by the presence of large pores with a mean diameter of 35 µm and small pores with a mean diameter of 1 µm (see the enlargement reported in Figure 2d). Differently from the PCL/starch blends, the distribution of the pores is homogeneous on the whole film surface, thanks to the similar solubility of scCO_2_ in the polymers. Figure 2d shows the presence of needle-like crystals due to the presence of MSG on the film surface.

**Figure 3:**
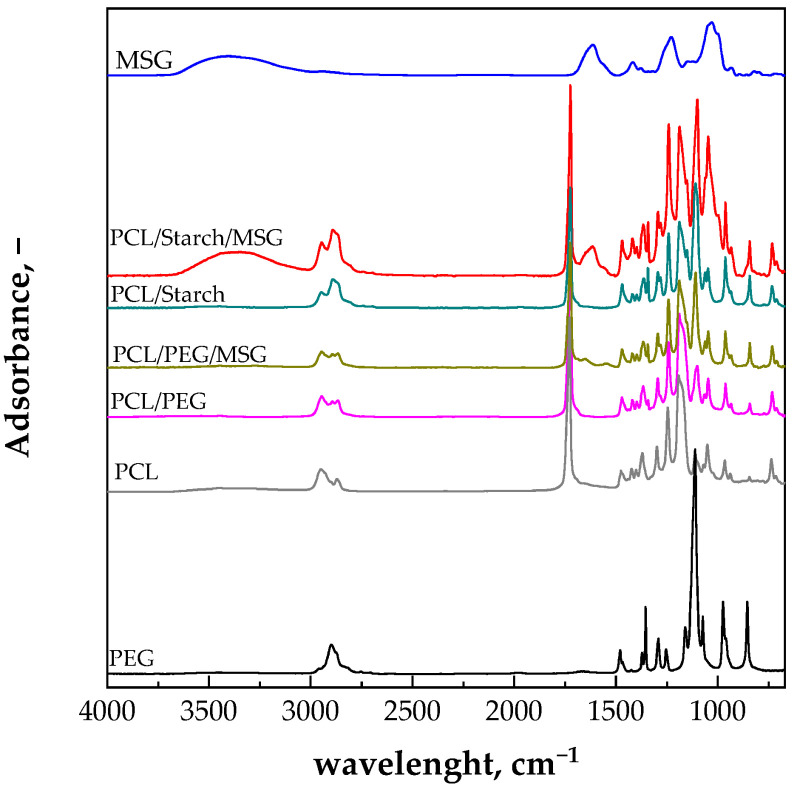
ATR spectra of the PCL/starch and PCL/PEG samples after the scCO_2_ impregnation with MSG. The ATR spectrum of MSG is also reported for comparison.

The presence of MSG on the surface of both the PCL/Starch and PCL/PEG films can be detected in the ATR analyses, shown in Figure 3.

MSG ATR spectrum shows a peak at 1615 cm^−1^ due to the carboxyl group. This peak is not present in the spectrum of the PCL/starch and PCL/PEG compression molded films. The presence of the peak at 1615 cm^−1^ in the spectrum of the scCO_2_ impregnated film confirms what was already observed in the SEM micrographs related to the presence of MSG on the film surface.

**Figure 4:**
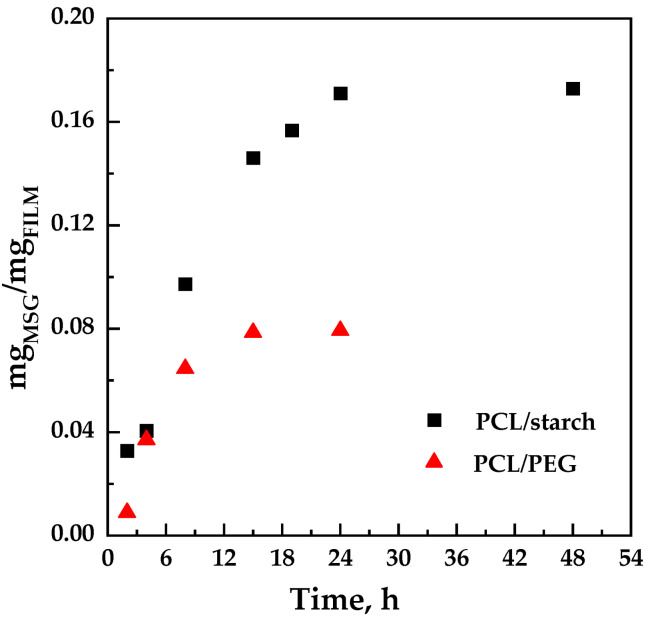
Adsorption kinetics for the scCO_2_ impregnated film of PCL/starch and PCL/PEG at 35 °C and 17 MPa.

The impregnation kinetics were determined at a fixed pressure of 17 MPa, and temperature of 35 °C, varying the contact time (from 2 to 48 h). To avoid considering the weight of the CO_2_ contained in the polymer matrix (which did not yet diffuse out of the polymer), the amount of impregnated MSG into the polymeric matrices was evaluated by UV/vis spectrophotometry. The values are reported in Figure 4 for the PCL/starch and PCL/PEG blends.

It can be observed that the quantity of impregnated MSG increased with the contact time up to reach a maximum value equal to 0.17 ± 0.02% and 0.08 ± 0.05% w_MSG_/w_FILM_ for PCL/starch and PCL/PEG films, respectively. Considering that starch does not form pores in the presence of CO_2_, while PCL and PEG tend to foam, a higher impregnation of MSG in the PCL/PEG system than in the PCL/starch system was expected. However, it should be considered that the quantity of active compound measured is not only due to the MSG impregnated inside the polymeric structure but also to the MSG that is deposited on the surfaces of the sample downstream of the depressurization.

**Figure 5:**
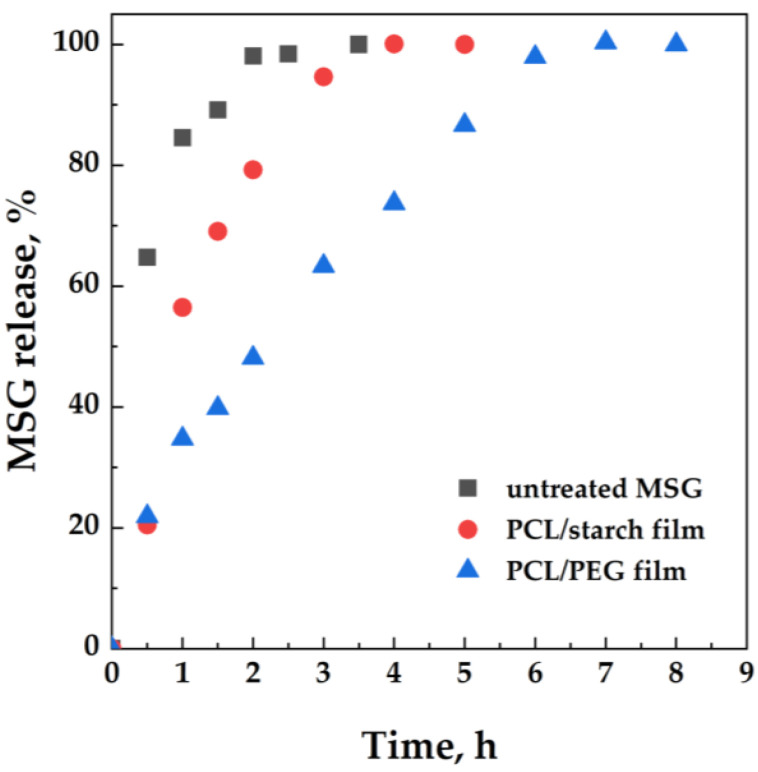
MSG release tests conducted in a Franz cell from PCL/Starch and PCL/PEG impregnated films. MSG dissolution profile is also reported for comparison.

It can be expected that the presence of MSG on the surface of both PCL/starch and PCL/PEG influences the release of the active molecule from the films. The release of MSG from the polymeric matrices has been analyzed using a Franz cell and a UV/vis spectrophotometer. Figure 5 shows the release profiles of MSG from both PCL/starch and PCL/PEG scCO_2_ impregnated films. The dissolution profile of the untreated MSG is also reported for comparison.

MSG rapidly dissolves in the release medium; particularly, an MSG percentage of about 62% dissolves in 30 min; 100% of MSG dissolves within 3.5 h. The impregnation of MSG in the polymeric matrices allows for the gradual release of the active molecules. The PCL/starch film releases 58% of MSG within 1 h, and the complete release of MSG from the polymeric film occurs within 4 h. The PCL/PEG film increases the release time; 60% of MSG is released in 3 h, and 100% of MSG is released within 6 h. The release rate for the two kinds of PCL films would depend on the behavior of the polymer in the presence of scCO_2_: scCO_2_ shows higher solubility in polymers instead of starch. This allows for adsorption of the active molecules in the inner layers of the polymeric matrix, thus decreasing the MSG release rate.

### 2.2. Electrospun Fibers

In this case, the films are composed of electrospun fibers forming membranes that can also be adopted for topical administrations. In these cases, scCO_2_ must transport the active molecule in the inner part of the membrane without compromising the original structure.

**Figure 6:**
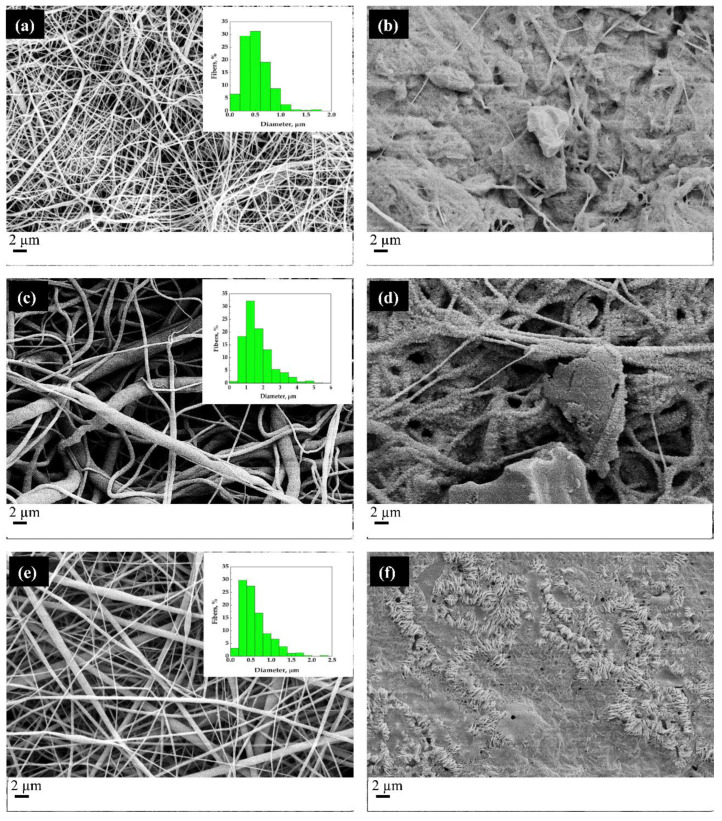
SEM micrographs of the electrospun fibers before the scCO_2_ impregnation process (**a**,**c**,**e**) and after the impregnation process conducted at 17 MPa and 35 °C (**b**,**d**,**f**). Particularly, (**a**,**b**) PCL/Gelatin, (**c**,**d**) PCL/PEG and (**e**,**f**) PCL/PVP are shown.

Figure 6 shows the SEM micrographs of the fibers, as obtained from the electrospinning process (images on the left) and the MSG-impregnated fibers (pictures on the right). The distributions of the diameters of the fibers are shown in boxes at the top right of the SEM images on the left.

The comparison between SEM micrographs of the electrospun fibers and the impregnated fibers makes evident that the scCO_2_ impregnation process induces a partial melting of the fibers; the structure of the fibers is altered by the supercritical processing under the chosen temperature and pressure conditions. Therefore, the operating conditions suitable for processing the films obtained by compression molding are not ideal for fibers. Indeed, in all the cases (PCL/gelatin, PCL/PEG, and PCL/PVP), the fibrous structure of the unprocessed sample is wholly lost in the MSG-impregnated sample. It should be emphasized that the polymers in question have relatively low melting temperatures and that the presence of supercritical carbon dioxide lowers them further. Indeed, it is well-known that the exposition of a semicrystalline polymer to a compressed fluid induced the melting point depression [44]; Fanovich and Jaeger [45] and Campardelli et al. [46] observed a decrease of the melting temperature from 62 °C (at atmospheric pressure) to 35 °C at pressures higher than 15 MPa. The images in the right column of Figure 6 suggest a fusion of the structure and subsequent resolidification with consequent loss of the microscopic fibrous structure.

**Figure 7:**
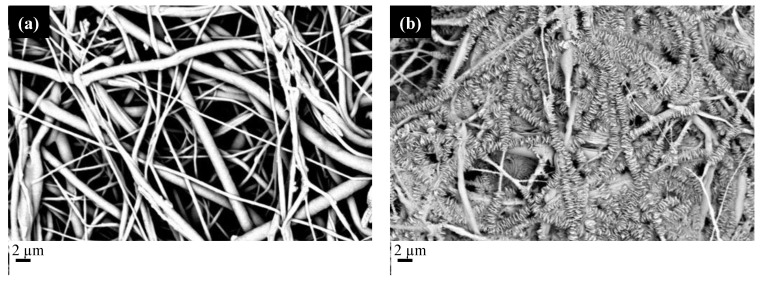
SEM micrographs of PCL/PVP fibers impregnated with MSG. The images are referred to two impregnation times (**a**) 2 h and (**b**) 15 h.

To reduce the plasticizing effect of carbon dioxide, the fibers were processed at lower pressure and temperature (15 MPa and 33 °C), hoping to obtain better results from a morphological point of view. Therefore, it was decided to carry out a first experimental campaign, placing the fibers in contact with supercritical carbon dioxide in the absence of MSG, to verify the effect of the presence of CO_2_ alone on the fibrous structure. Subsequently, the fibers were impregnated with MSG. Figure 7 shows the SEM micrographs of the PCL/PVP impregnated films adopting two impregnation times, 2 h and 15 h.

**Figure 8:**
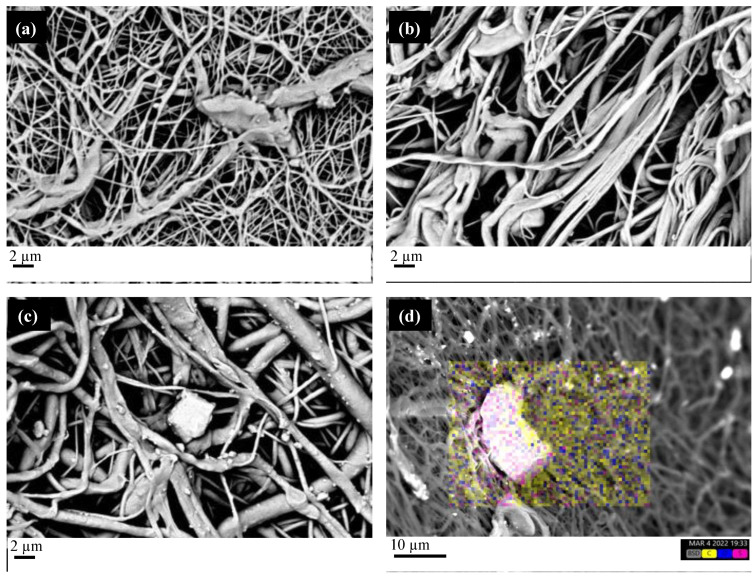
SEM micrographs of the electrospun fibers impregnated at 15 MPa, 33 °C, and 2 h impregnation time. (**a**) PCL/Gelatin; (**b**) PCL/PEG; (**c**) PCL/PVP. (**d**) EDS micrograph of the PCL/PVP electrospun fibers impregnated with MSG (the following colors are used for the elements: yellow for carbon, blue for oxygen, magenta for sulfur, characteristic of MSG).

The fibrous structure of the PCL/PVP film was not preserved when long impregnation times were adopted; the structure can be maintained if the impregnation time does not exceed 2 h. This finding can be ascribed to the crystallization of PCL during the impregnation process. Indeed, as already observed in the literature [46], scCO_2_ induces the decrease of the glass transition temperature, thus making molecular re-organization possible. If the impregnation time becomes comparable with the crystallization time, one can expect crystals to form, modifying the original structure of the electrospun fiber. PCL crystallization kinetics are very fast at the process temperature (33 °C) [47]; thus, the increase in the processing time induces an almost complete crystallization of PCL and a significant change in the membrane morphology. In particular, crystals grow from the center of the fiber toward the outer part forming disks. The PCL crystallization must be limited by selecting the proper impregnation time to avoid the aforementioned phenomenon. Figure 8 shows the SEM micrographs of the scCO_2_ impregnated films adopting an impregnation time of 2 h.

All the SEM micrographs show that the foamed structure characteristic of the electrospun fiber is preserved whatever the polymer composition, confirming that 2 h is suitable as impregnation time. Figure 8c clearly shows the presence of MSG crystal in the PCL/PVP film. The EDS analysis confirms such a presence, where sulfur, a characteristic element of MSG, can be detected in the bright area. Thus, scCO_2_ dissolves MSG, allowing for the transport of the active molecules in the inner part of the electrospun fibers, where MSG precipitates in the form of crystals without compromising the porous structure.

**Figure 9:**
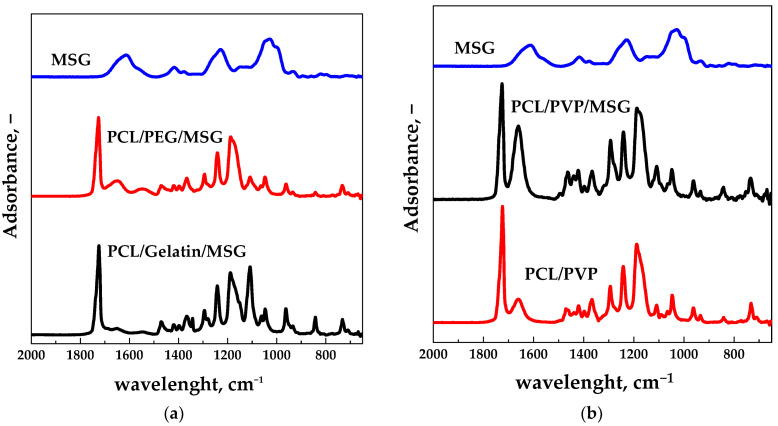
ATR spectra of the scCO_2_ impregnated films for different polymeric compositions: (**a**) PCL/PEG, PCL/Gelatin. (**b**) ATR spectra of the film PCL/PVP before and after impregnation. MSG ATR spectrum is also reported for comparison.

Figure 9a shows that the peak at 1615 cm^−1^, characteristic of the MSG molecule, is present in the spectra of the PCL/PEG and PCL/Gelatin impregnated films (the peak was not found in electrospun films). Figure 9b shows the ATR spectra of the film PCL/PVP before and after the impregnation process. The peak at 1615 cm^−1^ is also present in the spectrum of the electrospun PCL/PVP film (before scCO_2_ impregnation); however, its intensity is significantly higher in the MSG impregnated film. Thus, it can be hypothesized that the intensity increase is due to the MSG’s presence (as confirmed by the EDS analysis reported in Figure 8d).

Table 1 reports the MSG loadings, evaluated by UV/vis spectrophotometry, for PCL/PEG, PCL/Gelatin, and PCL/PVP electrospun films impregnated with 2 h processing time.

The PCL/PVP film shows more MSG in the polymeric matrix. The PCL/PEG film shows MSG content significantly higher than those evaluated for the PCL/PEG compression molded film (about 0.08 mg_MSG_/mg_FILM_, see Figure 4). This finding is due to the different impregnation mechanisms. In the case of the compression molded film, scCO_2_ must dissolve MSG, diffuse in the compact polymeric matrix, and release the active molecule; this generally leads to forming a foamed structure. In the case of the electrospun fibers, scCO_2_ must diffuse through a membrane-like structure (already porous) and release the active molecule. The diffusion through a membrane requires shorter times than the diffusion through a compact structure since the free volume is significantly higher in the first case [48].

**Figure 10:**
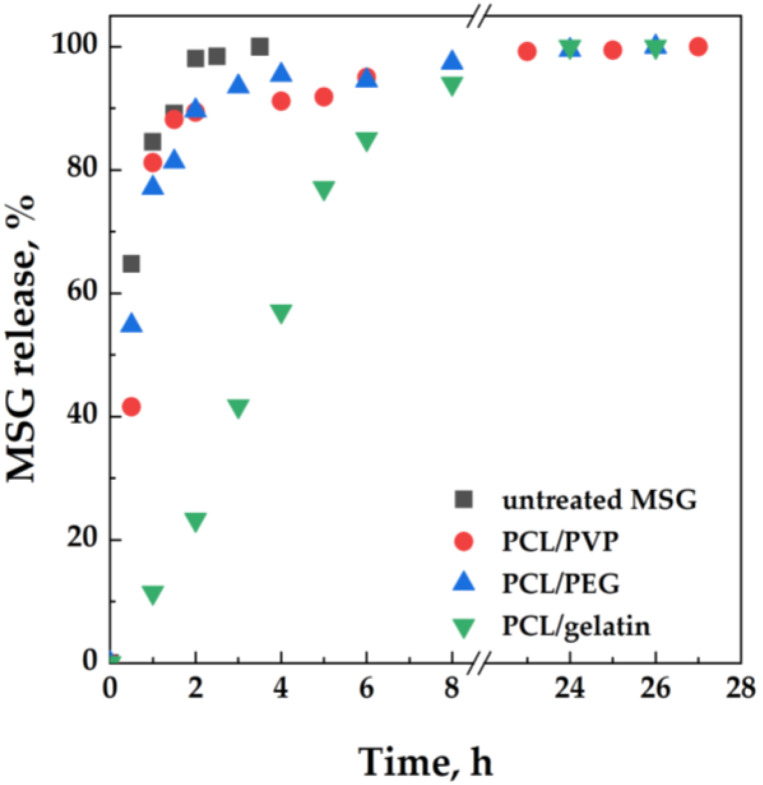
MSG release tests conducted in a Franz cell from PCL/PEG, PCL/Gelatin, and PCL/PVP impregnated films. MSG dissolution profile is also reported for comparison.

Figure 10 shows the MSG release profiles from the impregnated films, evaluated by adopting the Franz cell.

The MSG release profiles from PCL/PEG and PCL/PVP films are similar, with a significant burst effect at the beginning of the release test: about 82% of MSG is released from the polymeric matrix within 1 h. In the case of the PCL/Gelatin film, a prolonged release of the active molecule occurred: 60% of MSG is released within 4 h, and the complete release of the active molecule requires about 13 h. The prolonged release of MSG from the PCL/Gelatin matrix could be ascribed to the presence of MSG in the inner part of the film, whereas, in the case of PCL/PVP and PCL/PEG films, MSG is mainly present in the superficial layers, inducing the burst effect already discussed.

## 3. Materials and Methods

### 3.1. Materials

Polycaprolactone (PCL, average Mn ~80,000 Da by GPC), Polyethylene glycol (PEG, average Mn ~10,000 Da by GPC), Polyvinyl pyrrolidone (PVP, average Mn ~40,000 Da by GPC), Gelatin from bovine skin (CAS: 9000-70-8, average Mn ~261,000 Da) were bought from Sigma-Aldrich (Milan, Italy). Starch from maize (85652, 75% amylopectin, 25% amylose) was purchased from Fluka (Milan, Italy). Carbon dioxide (CO_2_, purity 99%) was purchased from Morlando Group S.R.L. (Sant’Antimo-NA, Italy). Sodium salt mesoglycan (MSG) was supplied by LDO (Laboratori Derivati Organici spa, Vercelli, Italy); it consists of heparin (40% low molecular weight in the range 6.5–10.5 kDa and 60% less than 12 kDa, sulphurylation degree 2.2–2.6), heparan sulfate (UFH-unfractionated heparin from 12 kDa up to 40 kDa; sulphurylation degree 2.6), and dermatan sulfate, deriving from epimerization of glucuronic acid of chondroitin sulfate (molecular weight 18–30 kDa, sulphurylation degree 1.3) with a total sulphurylation degree equal to 9.1.

Phosphate buffered saline solution (PBS, pH = 7.4) was prepared for the drug release study.

### 3.2. Melt Compounding and Compression Molding

Melt compounding was adopted for producing the following blends: PCL/PEG and PCL/Starch. Each polymer was dried for 2 h under vacuum at a temperature of 30 °C before any characterization and processing. Polymers were mixed by a counter-rotating twin-screw micro-compounder (HAAKE MiniLab II Micro Compounder, by Thermo Scientific, Waltham, MA, USA) with an integrated backflow channel. The materials were mixed at 65 °C and 30 rpm, with a backflow time of 5 min. The blends were used to obtain films via compression molding, adopting a molding press (Model C, Fred S. Carver Inc., Menomonee Falls, WI, USA), following the procedure: (a) pre-heating at 120 °C for 5 min, (b) compression-molding at 15 MPa for 2 min, and (c) cooling in air.

### 3.3. Electrospinning

The electrospun membranes were produced by dissolving, separately, PCL/Gelatin, PCL/PEG, and PCL/PVP (80/20 by weight) in a solvent mixture of CHCl_3_/CH_3_OH (75:25 *v*/*v*) at 10% *w*/*w* and mixed for 4 h at 40 °C (stirring rate = 300 rpm) to obtain a homogenous solution. After that, the polymeric solution was fed to a 5 mL syringe pump. The sets of electrospinning conditions are reported in Table 2 and optimized to produce beads-free fibrous membranes. Temperature and relative humidity (RH) were fixed for all the experiments (T = 25 °C and RH = 35%).

Climate controlled electrospinning apparatus (EC-CLI, IME Technologies, Gel-drop, The Netherlands) was used to produce fibrous membranes. A vertical setup was chosen to carry out the experiments. Electrospun membranes were obtained by using a one nozzle setup with a stainless-steel capillary (Ø_int_ = 0.8 mm).

### 3.4. Supercritical Impregnation

A homemade bench plant was used to perform the impregnation experiments. Experiments occur in a stainless still high-pressure vessel (NWA GmbH, Ahlen, Germany) with a 100 mL internal volume, closed on the bottom and on the top with two-finger tight clamps. The carbon dioxide is fed to the vessel by a diaphragm piston pump (Milton Roy, mod. Milroyal B, Pont-Saint-Pierre, France) after cooling through a cooling bath connected to the pump. Mixing is assured by an impeller mounted on the top cap and driven by a variable velocity electric motor. The operating pressure and temperature are measured by a digital gauge manometer (Parker, Minneapolis, MN, USA) and a K-type thermocouple with an accuracy of ±0.1 °C, respectively. The autoclave is heated through electrically controlled thin bands connected to a proportional–integral–derivative (PID) controller (Watlow, mod. 93, Toledo, OH, USA), which assures the thermal control into the vessel. At the exit of the autoclave, the CO_2_ flow rate is measured by a rotameter. Depressurization is performed by a micrometric valve (Hoke, mod. 1315G4Y, Spartanburg, SC, USA).

The solubility of mesoglycan in scCO_2_ was experimentally determined in previous papers in the range of pressure 12–18 MPa and a range of temperature equal to 35–60 °C [38,40], according to a well-established procedure [49,50]. The impregnation experiments were performed according to a static method [49]. A fixed amount of MSG (over its solubility in CO_2_ at the pressure and temperature conditions chosen for the experiment) is charged in a small container opened on the top and axially mounted on the impeller. The PCL-based film was placed on the bottom of the vessel inside a paper filter. After closing the finger-tight clamps, the CO_2_ was pumped, and the heating bands activated until the pressure and temperature chosen for the experiment were reached. Then, the CO_2_ supply was interrupted, the impeller was activated, and the system was left in batch for the time chosen for the experiment, after which the slow depressurization (at a constant flow rate equal to 0.1 MPa/min) was carried out. Once the atmospheric pressure was reached, the impregnated sample was recovered and characterized. Each impregnation experiment was repeated at least twice; the difference between the tests was less than 5%, probably due to the possible entrainment of the material through the filter paper during depressurization and to the deposition of non-impregnated MSG on the film’s surface.

### 3.5. Characterizations

A desktop scanning electron microscope (SEM) coupled with energy-dispersive X-ray spectroscopy (EDS) analysis (Phenom ProX with EDS detector (Phenom-World BV, Eindhoven, Netherlands)) was adopted for morphological characterization and element identification of films and electrospun fibers. All results were acquired using the ProSuite software integrated with Phenom Element Identification software, allowing for the quantification of the concentration of the elements present in the films, expressed in either weight or atomic concentration. Before the analysis, samples were covered with a thin film of gold by sputtering.

Fourier transform infrared spectroscopy was conducted on the materials adopted in this work to evaluate the amount of polymer and active molecule in each film and electrospun fiber. Attenuated total reflectance/Fourier transform infrared (ATR-FTIR) spectroscopy was conducted with a Perkin Elmer instrument (Spectrum 100, Perkin Elmer, Holdings Ltd., London, UK), in the range 4000–4500 cm^−1^, with 2 cm^−1^ resolution. ATR-FTIR spectra were deconvoluted by adopting the Peak-Analyzed plug-in of Microcal Origin (v 8.0) software, which allows the evaluation of the area below the characteristic peaks.

The amount of impregnated MSG into the polymeric matrices was evaluated by UV–vis spectrophotometry. MSG loadings were determined using a UV/vis spectrophotometer (model Cary 50, Varian, Palo Alto, CA, USA) at a wavelength of 206 nm. A total of 0.005 g of the film was placed in a filter and incubated in 10 mL of PBS at 200 rpm and 37 °C. The MSG loadings were measured by UV/vis analysis at the end of the release profiles, i.e., when all the MSG was released from the polymeric matrices. The absorbance was converted into MSG concentration using a calibration curve to check the weight increase of the sample measured at the end of the impregnation experiments.

Release studies were carried out at 37 °C using a vertical Franz diffusion cell 15 mm × 11 mL Type C in glass (Hosmotic SRL, Vico Equense, Napoli, Italy) coupled to the UV/vis spectrophotometer used for the loadings. The donor chamber was separated from the receptor chamber by a PVDF membrane having an outside diameter equal to 25 mm and a pore size of 0.45 μm. The receptor chamber was filled with 7.9 mL of PBS (pH 7.4) at 37 °C under constant stirring (500 rpm). Aliquots of 200 μL were withdrawn at fixed time intervals and replaced with equal volumes of fresh PBS.

## 4. Conclusions

The impregnation of MSG into films of PCL blends was attempted by adopting an innovative process assisted by scCO_2_. Particularly, blends of PCL and water-soluble polymers, PEG, PVP, Gelatin, and Starch, were employed to modulate the MSG release from the polymeric matrix, making the devices potentially suitable for topical administrations. Two different techniques were used to obtain the films: the first one is compression molding, producing compact and continuous structures, and the second one is electrospinning, producing membrane-like structures.

During the impregnation process of compression molded films, scCO_2_ has to solubilize the active compound, diffuse into the polymer matrix, and release the active compound. This process induced the formation of pores when compression molded films were adopted as a polymeric matrix. In these cases, a higher amount of MSG was loaded into the PCL/Starch matrix instead of the PCL/PEG matrix; however, the MSG release from the polymeric matrix is prolonged only in the case of PCL/PEG matrix. This finding was attributed to the behavior of the polymers composing the films in the presence of scCO_2_. scCO_2_ shows lower solubility in Starch than PCL and PEG. Thus, the impregnation process was less efficient in the case of the blend PCL/Starch: the amount of impregnated MSG was mostly distributed on the film surface, making the release faster.

scCO_2_ shows different behavior in the presence of membrane-like structures: it has to solubilize the active compound and release it into the membrane. The overall process is faster than what was previously described. As a result, the MSG loaded into the polymeric matrix was significantly higher than the one evaluated for the compression molded films. The MSG release was prolonged in the case of PCL/Gelatin film. Interestingly, in this case, the burst effect is significantly small, and the release of the active compound is gradual during the whole time. This makes the PCL/Gelatin film suitable for topical administrations.

## Figures and Tables

**Table 1:** MSG loadings measured by UV/vis analysis for the PCL/PEG, PCL/Gelatin, and PCL/PVP impregnated films.

Polymer Matrix	mg_MSG_/mg_FILM_
PCL/PEG	0.18 ± 0.01
PCL/Gelatin	0.12 ± 0.02
PCL/PVP	0.21 ± 0.01

**Table 2:** Process parameters of electrospun membranes.

Polymer Matrix	Voltage (kV)	Distance (cm)	Flow Rate (mL/h)
PCL/PEG	24	25	2
PCL/Gelatin	22	25	1.5
PCL/PVP	22	25	1.5

## Data Availability

Not applicable.

## Sample Availability

Samples of the compounds are available from the authors.
